# MicroRNA Regulation of the Small Rho GTPase Regulators—Complexities and Opportunities in Targeting Cancer Metastasis

**DOI:** 10.3390/cancers12051092

**Published:** 2020-04-28

**Authors:** Brock A. Humphries, Zhishan Wang, Chengfeng Yang

**Affiliations:** 1 Center for Molecular Imaging, Department of Radiology, University of Michigan, 109 Zina Pitcher Place, Ann Arbor, MI 48109, USA; 2 Department of Toxicology and Cancer Biology, College of Medicine, University of Kentucky, 1095 V A Drive, Lexington, KY 40536, USA; zhishan.wang@uky.edu

**Keywords:** Rho GTPases, Rho GTPase regulators, RhoGEFs, RhoGAPs, RhoGDIs, microRNAs, cancer, metastasis

## Abstract

The small Rho GTPases regulate important cellular processes that affect cancer metastasis, such as cell survival and proliferation, actin dynamics, adhesion, migration, invasion and transcriptional activation. The Rho GTPases function as molecular switches cycling between an active GTP-bound and inactive guanosine diphosphate (GDP)-bound conformation. It is known that Rho GTPase activities are mainly regulated by guanine nucleotide exchange factors (RhoGEFs), GTPase-activating proteins (RhoGAPs), GDP dissociation inhibitors (RhoGDIs) and guanine nucleotide exchange modifiers (GEMs). These Rho GTPase regulators are often dysregulated in cancer; however, the underlying mechanisms are not well understood. MicroRNAs (miRNAs), a large family of small non-coding RNAs that negatively regulate protein-coding gene expression, have been shown to play important roles in cancer metastasis. Recent studies showed that miRNAs are capable of directly targeting RhoGAPs, RhoGEFs, and RhoGDIs, and regulate the activities of Rho GTPases. This not only provides new evidence for the critical role of miRNA dysregulation in cancer metastasis, it also reveals novel mechanisms for Rho GTPase regulation. This review summarizes recent exciting findings showing that miRNAs play important roles in regulating Rho GTPase regulators (RhoGEFs, RhoGAPs, RhoGDIs), thus affecting Rho GTPase activities and cancer metastasis. The potential opportunities and challenges for targeting miRNAs and Rho GTPase regulators in treating cancer metastasis are also discussed. A comprehensive list of the currently validated miRNA-targeting of small Rho GTPase regulators is presented as a reference resource.

## 1. Introduction

Cancer progression is highlighted by changes in cancer cells that promote aggressiveness allowing cells to acquire a greater metastatic potential. Once cancer cells in the primary tumor gain the ability to invade the surrounding tissue, motile cells pass through the basement membrane and the extracellular matrix (ECM) penetrating into the lymphatic or vascular circulation. These motile cells travel through the circulatory system until they arrest at a different locations, extravasate through the vascular basement membrane and the ECM into the new environment where they gain epithelial characteristics and form a secondary or metastatic lesion. Because metastasis is the leading cause of mortality in cancer patients, recent research has focused on identifying and understanding the underlying mechanisms that contribute to metastasis. Numerous studies demonstrated that small Rho GTPases are key regulators of cell adhesion, migration and invasion, thus playing crucial roles in cancer metastasis (for reviews see [1,2,3]). It is well established that the activities of small Rho GTPases are tightly regulated mainly by the following four groups of regulators: guanine nucleotide exchange factors (GEFs), GTPase-activating proteins (GAPs), guanosine diphosphate (GDP) dissociation inhibitors (GDIs) and guanine nucleotide exchange modifiers (GEMs) [4,5,6,7]. However, much less is known about how the activities of small Rho GTPase regulators are regulated.

Although elucidating the underlying mechanisms of cancer metastasis has been the focus for many years, the connection between microRNAs (miRNAs), a family of small non-coding RNAs, and Rho GTPase regulators has only recently become a focused topic in cancer metastasis studies. There is a growing body of evidence revealing the critical involvement of miRNAs in the tight spatiotemporal regulation of actin-based physiology. Moreover, depending on the specific context, miRNAs can have a tumor suppressive or oncogenic role in cancer. We understand that miRNAs can directly regulate the expression of Rho GTPases and this was reviewed elsewhere [8]. In this review, we focused on recent exciting findings showing that miRNAs play important roles in regulating Rho GTPase regulators (RhoGEFs, RhoGAPs, RhoGDIs), eventually affecting small Rho GTPase activities and cancer metastasis. A comprehensive list of the currently validated miRNA-targeting of small Rho GTPase regulators is presented.

## 2. MicroRNA Biogenesis and Function

Although the basic features of microRNA biogenesis and its mechanism of action were established over a decade ago [9,10,11], subsequent years have shown a vast accumulation of new information that has not only deciphered the mechanistic details, but has also demonstrated that miRNAs are key regulatory hubs for cancer. Here, we provide only a brief introduction to miRNA biogenesis and function for context as we discuss their direct role in modulating mechanisms that contribute to cancer progression (we have previously reviewed miRNA biogenesis in more detail [12,13,14]).

**Figure 1:**
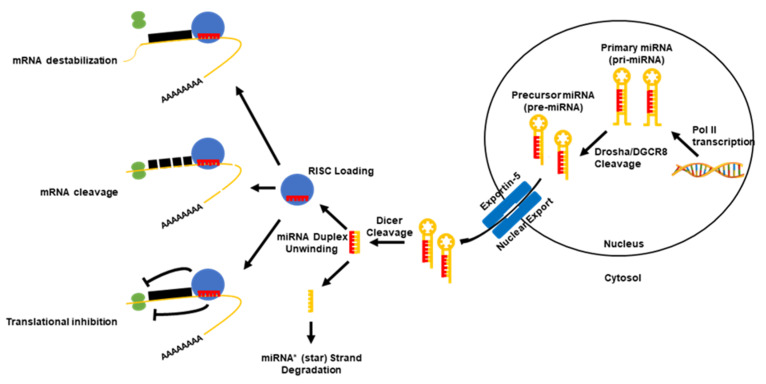
Canonical microRNA (miRNA) biogenesis pathway. Canonically, the miRNAs are transcribed in the nucleus via their own promoters or their host gene promoters by RNA polymerase II or III. This results in the formation of a primary miRNA (pri-miRNA) transcript, which can range from hundreds to thousands of nucleotides long. After a polyadenylation and capping event, pri-miRNAs undergo a microprocessing cleavage event by a ribonuclease (RNase) III type enzyme, Drosha, and its binding partner DiGeorge syndrome critical region gene 8 (DGCR8) resulting in a ~60–120 nucleotide-long precursor miRNA transcript (pre-miRNA). Pre-miRNAs are then exported out of the nucleus by the karyopherin exportin-5 to the cytoplasm, where the RNase II enzyme, Dicer, processes the transcripts to form a miRNA duplex. The unwinding of the miRNA duplex occurs and one strand is usually degraded (miRNA* (star) strand), while the other (mature miRNA) is loaded into the RNA-induced silencing complex (RISC). The RISC probes for targets of the miRNA in the genome. Once bound to a target mRNA, the RISC may induce a negative expression of the mRNA in three ways: (1) mRNA destabilization and degradation, (2) mRNA translational inhibition, or (3) mRNA cleavage. The path at which the mRNA is regulated depends upon multiple factors of the mature miRNA.

MicroRNAs (miRNAs or miRs), are a subclass of small (~21–23 nucleotides) non-coding RNA molecules that negatively regulate protein-coding gene expression. In terms of biogenesis (Figure 1), a functionally mature miRNA is derived from the cleavage of a double-stranded ~70 nt RNA hairpin precursor in the cytosol. These miRNA precursors are typically located either within the introns of a host protein-coding gene or in intergenic regions, and are transcribed in the nucleus by either RNA polymerase II or III. However, the cases in which miRNA precursors were found within the exons of transcripts and in antisense transcripts have been reported [15,16]. Once excised from the precursor RNA hairpin, a mature miRNA is then loaded into the RNA-induced silencing complex (RISC), where miRNAs are then able to negatively regulate the expression of target genes. Functionally, miRNAs elicit this negative regulation typically by imperfect base pairing with the 3′ untranslated region (3′UTR) of the target messenger RNA (mRNA) through the miRNA seed sequence. The seed sequence is the second to eighth nucleotide at the 5′ end of a mature miRNA. The binding of a miRNA to its target mRNAs can induce mRNA degradation, translational inhibition or direct cleavage, depending on the sequence complementarity [11,17,18,19]. The expression of miRNAs can be regulated through interactions with and the modifications of their promoters. In addition to the promoter-mediated control of expression, miRNA function can be controlled through the binding and sequestration of the mature miRNA in the cytosol by long non-coding RNAs (lncRNAs) as well as circular ncRNAs (circRNAs), termed competing endogenous RNAs (ceRNAs) [20]. Due to their direct role in regulating the gene expression of most of the human genome [16,21,22], miRNAs are directly involved in almost all aspects of cellular functions. Specifically, the cellular pathways that underlie cancer progression are regulated by either oncogenic or tumor suppressive miRNAs [12,23]. This suggests that miRNAs are central regulatory elements and represent a promising avenue for therapeutic intervention.

## 3. The Small Rho GTPases

Ras small GTPases are a superfamily of monomeric hydrolases that are found in all eukaryotic cells and function similarly to the α-subunits of heterotrimeric G proteins. Small GTPases act as molecular switches to facilitate cell activities including proliferation, morphology change, adhesion, migration, invasion and nuclear or vesicular transport, among others. This molecular switching is driven by binding and hydrolyzing GTP, leading to the transition of small GTPases between three conformational states; 1) the GDP-bound, 2) the GTP-bound and 3) the empty state that transiently exists between the replacement of GDP with GTP in the guanine nucleotide binding site [24]. The three main areas of the GTPase that change between GTP- or GDP-bound states are referred to as the phosphate binding loop (P-loop), switch 1 (residues 30–40, also known as the effector loop) and switch 2 (residues 60–76) [25,26], all of which reside within the GTP-binding site of the GTPase. The GDP-bound state is generally considered inactive (“off”), while the GTP-bound form is the active (“on”) form which allows GTPases to move to the cell membrane region and interact with downstream effectors.

The small Rho GTPase family is one of the five originally classified major subfamilies of the Ras small GTPase superfamily [27]. It consists of 20 small (190–250 residues) molecules which control the cytoskeleton and cell morphology specifically by regulating actin dynamics (Table 1). They share ~30% sequence identity with the other Ras superfamily proteins and between 40–95% sequence identity within the subfamily. In addition to containing sequence motifs common to all Ras small GTPases, what structurally separates Rho small GTPases from other proteins in the Ras superfamily is the insertion of 9–12 residues, after residue 122 located between the fifth β-strand and fourth α-helix within the GTPase domain [28,29]. The majority of Rho GTPases undergo C-terminal post-translational modifications by isoprenoid lipids or palmitate fatty acids [30,31], which help localize their subcellular localization and association with membranes or organelles. In addition to modifications to their C-terminal, Rho GTPases are also directly regulated by GTPase-activating proteins (GAPs), guanine nucleotide exchange factors (GEFs), GDP dissociation inhibitors (GDIs) and guanine nucleotide exchange modifiers (GEMs) discussed later in this review. Of the 20 members of the Rho GTPase subfamily (Table 1), the best characterized Rho molecules are RhoA, Rac1, and Cdc42. RhoA promotes actin–myosin contractility and thus controls stress fiber and focal adhesion formation and turnover. Rac1 drives actin polymerization for the formation of membrane ruffling and lamellipodia, or the large projection at the leading edge of the migrating cell. Cdc42 regulates the formation of filopodia, which are actin-rich, finger-like projections that exude from the lamellipodia at the leading edge of the migrating cell.

## 4. Regulators of the Small Rho GTPases

**Figure 2:**
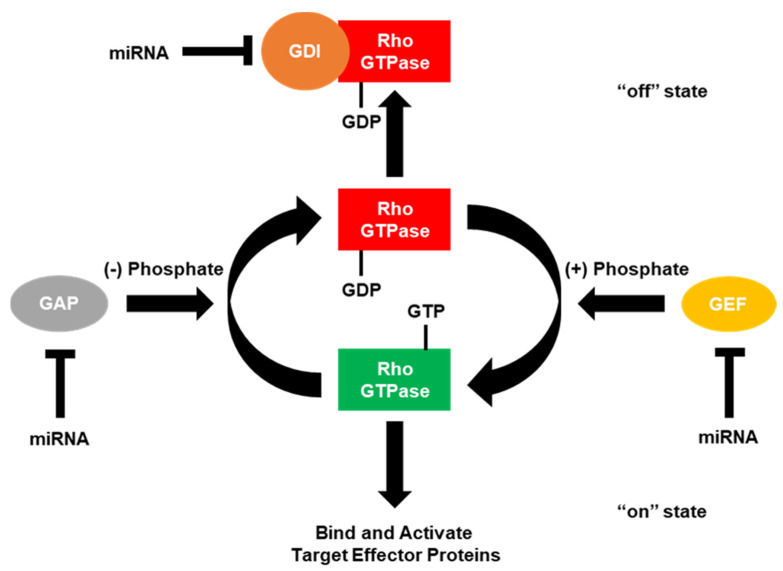
GTPase-activating proteins (GAPs), guanine nucleotide exchange factors (GEFs) and guanosine diphosphate (GDP) dissociation factors (GDIs) regulate small Rho GTPases. Small Rho GTPases are known as molecular switches due to the fact they cycle between “on” (GTP-bound) and “off” (GDP-bound) states. In the GTP-bound state, small Rho GTPases are able to regulate intracellular signaling cascades by binding and activating effector molecules. This signaling can be terminated by the intrinsic GTPase capability (GTP to GDP) of small Rho GTPases, which is enhanced by the interaction with GTPase-activating proteins (GAPs). While in the GDP-bound state, Rho GTPases can also interact with guanosine nucleotide dissociation inhibitors (GDIs), which sequester the small Rho GTPase and do not allow for the GDP to be exchanged for GTP. In order for GDIs to release the small Rho GTPase, a release factor must be present. Conversely, in the GDP-bound state, small Rho GTPases are unable to regulate downstream signaling, but can be reactivated by exchanging GDP for GTP, facilitated by the interaction with a guanine nucleotide exchange factor (GEF). Current literature has demonstrated that miRNAs can directly bind and downregulate the expression of RhoGAPs, GEFs, and GDIs to regulate cancer progression.

The “on” and “off” states of the Rho GTPases can be accelerated by the interaction with certain regulators of G-protein signaling (Figure 2) [4,5,6]. GTPase-activating proteins (GAPs) accelerate the Rho GTPases intrinsic phosphatase capability, putting the GTPase into the “off” state. Conversely, guanine nucleotide exchange factors (GEFs) activate Rho GTPases by rapidly exchanging GDP with the GTP. GDP dissociation factors (GDIs) also act to put the Rho GTPases into the “off” state by binding and sequestering Rho GTPases. Since the number of GAPs and GEFs outnumbers the number of Rho GTPases by over 3 to 1, many of these GAPs, GEFs and GDIs target the same Rho GTPase. However, some of these GAPs, GEFs and GDIs have been shown to be specific for a single Rho GTPase over the others. Moreover, these regulators of small G-protein signaling have been shown to be regulated by the small Rho GTPases themselves.

### 4.1. GTPase-Activating Proteins (GAPs)

The Rho GTPase-activating proteins (RhoGAPs) are one of the major regulators of Rho GTPases found in all eukaryotes. They are defined by the presence of a conserved 150 residue RhoGAP domain, which is distinct from GAPs for other classes of GTPases. This domain consists of nine α helices and a conserved arginine residue in a loop structure [47]. The RhoGAP domain gives RhoGAPs their function because it is sufficient for the binding to GTP-bound Rho proteins as well as accelerating their GTPase activity. Currently, there are over 60 RhoGAPs reported in humans (Table 2), far outnumbering their Rho GTPase substrates. This suggests that each RhoGAP may play a specialized role in regulating the multiple GTPase activity, location, or effector association and mandates the tight control of RhoGAP activity so that Rho GTPases are not always in the “off” state. Traditionally, GAPs were thought of as tumor suppressors. However, as some recent work has demonstrated, the overexpression of RhoGAPs in some cancers [48,49,50] and the interaction between the Rho GTPases and RhoGAPs may be more complex and context-dependent than originally thought. Expanding this to miRNAs, this would also suggest that miRNAs that directly target RhoGAPs also play a context-dependent role in cancer progression.

Insight into how RhoGAPs facilitate the intrinsic GTPase activity of Rho GTPases stems from structural work on Ras GTPase-activating proteins (RasGAPs). This is because even though the GAP domains differ, the tertiary structure and the fundamental GTPase-activating mechanism are similar to that of the RasGAPs [260,261]. Firstly, the RhoGAP interacts with the P-loop, switch 1 and the switch 2 regions of the GTP-binding site. This causes a slight rotation of the GTPase, placing the arginine residue of the RhoGAP directly into the active site of the Rho GTPase [262,263]. This not only begins to stimulate the catalysis of GTP, but also stabilizes any charges developed during the transitional state [264]. The inserted arginine interacts with Gln61 in the switch 2 region of the GTPase. The Gln61 residue is important because it is responsible for the interaction with a water molecule used to stimulate the intrinsic GTPase catalytic activity. This water-mediated nucleophilic attack is performed on the terminal ɣ phosphate of a GTP molecule, producing GDP and an inorganic phosphate. Although it is not well known how RhoGAPs choose their specific Rho GTPase target, it has been suggested that this is achieved by recognizing sequences in the α3 helix of the Rho GTPase [265,266].

### 4.2. Guanine Nucleotide Exchange Factors (GEFs)

Rho guanine nucleotide exchange factors (RhoGEFs) are directly responsible for the activation of Rho GTPases by catalyzing the exchange of GDP for GTP. Some RhoGEFs display specificity toward a single Rho GTPase, while others exhibit more promiscuity. As in the case of RhoGAPs, the highly controlled regulation of RhoGEF activity is paramount to ensure that Rho GTPases are not always in the “on” state. This regulation and specificity of RhoGEFs is determined by regulatory mechanisms such as posttranslational modifications, having differing sensitivities to lipids, direct binding to surface receptors, and an association with specialized complexes [267,268,269]. Based upon their functions, this would suggest that aberrant RhoGEF regulation, such as increased gene expression or mutations causing constitutive activation, is a main driver behind cancer progression. Indeed, RhoGEFs are positive regulators of cancer progression, where their increased expression drives cancer cell migration, invasion, adhesion and metastasis. Therefore, this suggests that miRNAs that directly target RhoGEFs function as tumor suppressors. However, this idea has been challenged recently where a RhoGEF (ARHGEF10) was shown to function as a tumor suppressor in pancreatic ductal adenocarcinoma [270].

There are two subfamilies of RhoGEFs: the diffuse B-cell lymphoma (Dbl) and dedicator of cytokinesis (DOCK) families. The 73 members of the Dbl family (Table 2) share a ~200 residue catalytic Dbl homology (DH) domain immediately preceding an adjacent regulatory ~100 residue pleckstrin homology (PH) domain [271]. However, some family members possess tandem DH and PH domains or completely lack a PH domain [272]. They also differ significantly in the N- and C-terminal sequences, which is used to regulate the intrinsic RhoGEF catalytic activity, localization, or complex association as described above. Additionally, it should be noted that unlike humans, plants do not possess Dbl RhoGEFs [273]. Functionally, the DH domain of Dbl RhoGEFs is responsible for facilitating the exchange of GDP for GTP by stabilizing switch 1, the remodeling of the alanine near the Mg2+ binding site in switch 2, and stabilizing the P-loop of the Rho GTPase [6]. Dbl RhoGEFs have been shown to act on RhoA, Rac1, and Cdc42. Conversely, the DOCK RhoGEFS (Table 2) are characterized by a conserved Dock-homology region-2 (DHR2) domain that serves as the catalytic domain and the DHR1 domain that locates them to specific membranes. What makes these RhoGEFs unique is that the DHR2 domain stabilizes the switch 1 of the Rho GTPase using interactions not seen in other typical RhoGEFs. DOCK proteins also utilize a valine residue to help dissociate the bound GDP, but does not distort switch 2 of the GTPase like Dbl family members [6]. DOCK proteins are shown to act primarily as RhoGEFs for Rac1 and Cdc42, but not RhoA [274,275].

### 4.3. GDP Dissociation Inhibitors (GDIs)

In addition to RhoGAPs and RhoGEFs, Rho GTPase GDP dissociation inhibitors (RhoGDIs) perform a unique function in the regulation of Rho GTPases. Characterized by a conserved ~60 residue N-terminal domain, RhoGDIs prevent Rho GTPases from activation and the subsequent interactions with downstream effectors through three different mechanisms: (1) binding and sequestering Rho GTPases in the inactive, GDP-bound form preventing activation by RhoGEFs. (2) binding and sequestering Rho GTPases in the active, GTP-bound form preventing the hydrolysis of GTP by either the intrinsic or the RhoGAP-stimulated GTPase activity, and (3) modulating the cycling of Rho GTPases between the cytosolic and membrane localization [276,277]. These biochemical functions mean that RhoGDIs have dual roles in the cell; they form soluble complexes with GDP-bound Rho GTPases in the cytosol, but also monitor Rho GTPases at the site of action on membranes.

To elicit their effects, RhoGDIs recognize the isoprenoid geranylgeranyl lipids at the C-terminus of the Rho GTPase [276,277]. Once bound to the Rho GTPase, the N-terminal domain of the RhoGDI interacts with the switch 1 and switch 2 of the Rho GTPase, restricting the spatial flexibility needed to exchange GDP or hydrolyze GTP. In contrast to the large number of RhoGAPs and RhoGEFs, only three RhoGDIs have been identified in mammals (Table 2). RhoGDIα (also known as RhoGDI1) is the most commonly found and ubiquitously expressed RhoGDI and it is able to form complexes with most members of the Rho family [250,253,278]. RhoGDIβ (also known as RhoGDI2) is predominantly expressed in hematopoietic cells [279,280], but its dysregulation is also found in certain cancer types [281,282,283,284]. It can interact with several Rho GTPases, but the affinity for complexing is 10–20 fold lower than that of RhoGDIα [256,285]. Lastly, RhoGDIɣ (also known as RhoGDI3) is preferentially expressed in the brain, lung, kidney, testis and pancreas [258,259], and is targeted to the Golgi complex through its N-terminal domain where it predominantly interacts with RhoB and RhoG [257,258]. The dysregulation of the RhoGDIs is linked to cancer cell migration, invasion, and metastasis [286,287], but the downstream effects of the altered RhoGDI expression seems to be context and cancer-type dependent. Additionally, it has been shown that RhoGDI mRNA can interact with regulators critical to the miRNA biogenesis, stability and activity [288], suggesting a more diverse role for RhoGDIs in cancer cells.

### 4.4. Guanine Nucleotide Exchange Modulators (GEMs)

In addition to GAPs, GEFs, and GDIs, another family of GTPase regulators has recently been identified. The guanine nucleotide exchange modulators (GEMs) are unique because they function as both a GEF and a GDI depending on context [7,289]. The members of this family are characterized by a ~30 residue domain that directly binds to G proteins. GEMs share little sequence homology between family members and act as central regulators to diverse G protein signaling cascades. The prototypical GEM family member is Girdin (for Girder of actin [290], also known as GIV [291], HkRP1 [292], or APE [293]), which is a multi-domain cytosolic protein that was identified to regulate the actin cytoskeleton during cell migration. Although it has been shown to drive actin cytoskeletal remodeling and bind to α-subunits of heterotrimeric G proteins like other GTPase regulators, these studies were not performed using human Rho GTPases. Therefore, it is not yet known if GEMs can directly modulate Rho GTPases activity.

## 5. MiRNAs Target RhoGAPs, RhoGEFs and RhoGDIs to Regulate Rho GTPase Activities and Cancer Progression

In addition to directly targeting Rho GTPases themselves [8], miRNAs are also directly involved upstream by targeting the modulators of Rho GTPase activity. This section will focus on miRNAs that have been identified to directly bind to the 3′UTR of RhoGAPs, RhoGEFs and RhoGDIs in cancer. The decreased expression of these Rho GTPase modulators has context-dependent effects on processes of cancer progression which we review here. A full list of the miRNAs that have been shown to reduce the expressions of RhoGAP, RhoGEF and RhoGDI mRNA and/or protein expression is found in Table 3.

### 5.1. MiRNA Targeting of RhoGAPs

Many studies including ours have shown that active Rho GTPases promote cancer cell survival, proliferation, migration and tumor metastasis [426]. Therefore, RhoGAPs, which are negative regulators of Rho GTPases, are usually considered to have tumor-suppressive functions [427]. However, data elucidating the physiologic function of the miRNA targeting of RhoGAPs in cancer currently show the most context-dependent effects of the three groups of Rho GTPase regulators. For example, it was found that the enforced expression of miR-34a in lung cancer cells [296] or miR-509 in primary osteosarcoma cells [297] reduced the growth and migration in vitro, and tumor invasion and metastasis in mice through targeting ARHGAP1. Furthermore, decreased ARHGAP1 expression by miR-509 sensitized cells to cisplatin, a commonly used chemotherapeutic [297]. However, Satterfield et al. found that the reduction of ARHGAP1 by miR-130b in Ewing sarcoma induced the cancer cell growth, migration and invasion in vitro, and promoted lung colonization when injected into the tail vein of Rag2^−/−^ mice [298]. In a different study, MDA-MB-231 breast cancer cells stably expressing miR-940 injected into the calvarial or tibial bones of BALB/cAJcl-nu/nu mice resulted in tumors with enhanced osteoblastic lesions [299]. This study determined that ARHGAP1 was a direct target of miR-940 and the reduction of ARHGAP1 led to an increase in osteoblastic lesions.

The context-dependent effects of the miRNA targeting of ARHGAP5 have also been found. Since, Wang et al. previously found that miR-486-5p was significantly downregulated in non-small cell lung cancer (NSCLC), and they next aimed to identify the effects of miR-486 on NSCLC cancer progression [300]. The ectopic addition of miR-486-5p into A549 and H157 NSCLC cell lines resulted in the significant inhibition of cell growth as well as cell migration and invasion as determined by a Transwell migration assay. A tail vein injection of the cells transfected with miR-486-5p demonstrated that miR-486-5p could reduce colonization and growth in the lungs. Mechanistically, they determined that the oncogenic ARHGAP5 was a direct target of miR-486-5p, and the silencing of ARHGAP5 recapitulated the phenotypes of increased miR-486-5p expression [300]. Conversely, in two separate studies the induction of miR-494 by ionizing radiation or miR-744 by lactic acid enhanced the cell motility and invasion through the targeting of ARHGAP5 [301,302]. Interestingly, instead of decreasing the expression of ARHGAP5, it was shown that miR-744 bound directly to the promoter and increased the expression of ARHGAP5 to drive these processes of cancer progression [302,303]. This suggests a more complex regulatory mechanism of miRNAs with ARHGAP5 in cancer.

The miR-200 family is well known to regulate many aspects of cancer progression [12]. Therefore, it is not surprising that works have identified the effects of the miR-200 family on RhoGAPs. Deleted in liver cancer 1 (DLC1, also known as ARHGAP7) is typically thought of as a tumor suppressor, however, studying the miR-200 family discovered the inconsistent effects of targeting on cancer progression. In colorectal cancer, Wu and colleagues found that miR-141 drove cancer progression [306]. In addition to miR-141 inversely correlating with DLC1, the transient or stable expression of miR-141 in Lovo colorectal cancer cells promoted cell growth, migration and invasion, as well as tumor growth. They found that the miR-141-induced cell growth was accompanied by an increase of cells in the G2/M phase and a decrease in the G0/G1 phase of the cell cycle, suggesting that miR-141 could promote cell cycle progression. Xiao et al. found that the expression of another miR-200 family member, miR-429, similarly increased cell growth by directly targeting DLC1 in non-small cell lung cancer (NSCLC) [308]. In both studies, the expression of DLC1 lacking the 3′UTR in miRNA-expressing cells was able to overcome the oncogenic effects, confirming the oncogenic mechanism of miR-141 and -429 through targeting DLC1. Conversely, miR-200a, -200b and -200c were shown to suppress cancer progression [310]. In direct contrast to the study with miR-429 [308], the re-expression of another miR-200 family members not only resulted in an increase in E-cadherin, characteristic of a non-migratory and invasive epithelial cell, but also reduced the gene expression profile of a metastatic lung cell line in response to the direct targeting of DLC1 in NSCLC [310]. Moreover, Ibrahim et al. showed that the miR-200c-mediated DLC1 mRNA inhibition led to enhanced growth and colony formation, but reduced migration and invasion in serous ovarian cancer [311]. Since members of the miR-200 family share the same targeting seed sequence [12] and conflicting results are even obtained within the same cancer type [308,310], defining contexts in which environmental factors contribute to the miRNA regulating effects of cancer progression warrants further investigation.

Although the targeting of the aforementioned RhoGAPs generated both controversial and context-dependent results, some RhoGAPs maintain a more consistent role in cancer. Examples of this include; (1) the reduction of ARHGAP18 expression by the stable expression of either miR-153 or miR-200b inhibited the cancer cell migration and metastasis in liver and breast cancer cell lines [50,318], (2) the targeting of RACGAP1 by miR-192, -204, or -4324 induced the cell cycle arrest and reduced migration and invasion in osteosarcoma [356], pancreatic [357] and bladder cancer [358], respectively, and (3) the targeting of ARHGAP37 (STARD13) by miR-9 or miR-125b drove cancer cell growth, migration, invasion and metastasis [323,324,325,326]. Together these data suggest that based upon the currently available literature, some RhoGAPs act specifically as oncogenes or tumor suppressors.

IQGAP1 is also targeted by miRNAs, and although it is a scaffolding RhoGAP that does not possess intrinsic GTPase-activating capabilities, it has been shown to function as an oncogene in cancer. The study by Furuta and colleagues found that miR-124 and miR-203 were frequently methylated in hepatocellular carcinoma (HCC), and inversely correlated with patient prognosis [330]. The ectopic expression of miR-124 or miR-203 both not only suppressed hepatocellular carcinoma cell growth, but also induced cell cycle arrest and apoptosis in part by the direct targeting of IQGAP1. The regulation of IQGAP1 expression by miR-124 has also been identified in glioblastoma [331] and in endometrial cancer [332]. In both of these studies, the suppression of IQGAP1 either by direct repression or the ectopic expression of miR-124 blunted cell migration and invasion. These data suggest miR-124 as a tumor suppressor in multiple cancer types. Furthermore, Sun et al. found that miR-506 was inversely correlated with patient prognosis and the tumor stage in breast cancer [333]. In addition to the reduction of cell growth, invasion and adhesion, the expression of miR-506 also reduced MAPK/ERK signaling. This group found that miR-506 suppressed these functions and pathways by targeting IQGAP1, and the expression of IQGAP1 lacking the 3′UTR rescued the effects of miR-506 on breast cancer progression [333]. Collectively, these data suggest that although IQGAP1 lacks the typical GTPase-activating function, the dysregulation of IQGAP1 regulation is a critical step in cancer progression for different cancer types.

### 5.2. MiRNA Targeting of RhoGEFs

Since RhoGEFs are positive regulators of Rho GTPases, they are generally thought to have oncogenic functions in cancer. Therefore, miRNAs that directly target RhoGEFs function as tumor suppressors. Current literature supported this as the reduction of RhoGEF expression by miRNAs generally blunts cancer progression. One of the most well known Dbl family RhoGEFs is the son of sevenless 1 (SOS1), which not only activates Rho GTPases, but is also known to scaffold and activate Ras [428]. Due to its central function in cancer progression, many studies have found that the downregulation of SOS1 expression blocks many aspects that define cancer. In support of this, studies have found that both miR-20b and miR-4728 expression levels were significantly reduced in papillary thyroid cancer (PTC) compared to adjacent normal thyroid tissue, but that low levels of these miRNAs correlated with worse patient prognosis [383,389]. The ectopic expression of either of these miRNAs inhibited PTC cell growth, migration and invasion. In support of the critical function of SOS1 in Ras-mediated signaling, the expression of miR-20b and miR-4728 significantly blunted MAPK/ERK signaling by directly targeting SOS1. It was also found that the expression of SOS1 was able to overcome miRNA-mediated inhibition on growth, migration, invasion and signaling [383]. SOS1 can also promote bone metastasis through the direct modulation of breast cancer stem cell (CSC) migration and invasion [388]. Lin et al. found that miR-628 was significantly downregulated in bone metastatic breast cancer cells compared to the cells of the primary tumor. As CSCs are the major driving force behind metastasis, they next determined the effects of miR-628 expression on the functions of CSCs. The ectopic expression of a miR-628 mimic in CD44^+^/CD24^−^ breast CSCs significantly reduced cell migration and invasion as assayed by a Transwell assay. Additionally, the miR-628 mimic also reduced the expression of mesenchymal markers, vimentin and Snail, and increased the E-cadherin epithelial marker, suggesting a non-migratory phenotype. This study determined that miR-628 attenuates CSC migration and invasion through the direct targeting of the SOS1 3′UTR, and that the SOS1 expression was able to overcome the inhibitory effects of miR-628 on migration and invasion [388]. These data collectively demonstrate that SOS1 is an oncogene and miRNAs that directly target SOS1 act as tumor suppressors.

Another the well studied RhoGEFs is T-lymphoma invasion and metastasis-inducing protein 1, or TIAM1. Although originally identified as a Rac-specific GEF, studies have also identified GEF effects on RhoA and CDC42, albeit to a less efficient extent [226], where TIAM1 operates as a potent oncogene. Two members of the miR-10 family (miR-10a and miR-10b) have been shown to function as tumor suppressors by directly targeting TIAM1 [391,392,393,394]. The expression of these two miRNAs were found to be significantly reduced in tumor tissue compared to the matched normal tissue [391,393,394]. The stable expression of these two miRNAs inhibited Rac activation, cell growth, migration and invasion, as well as induced apoptosis by regulating TIAM1 expression. Furthermore, these results translated to mice as the expressions of miR-10a and miR-10b were found to reduce tumor growth and metastasis in esophageal squamous cell carcinoma [391] and cervical cancer [393], respectively. It was found that miR-10b was epigenetically silenced by DNA methylation [393,394], suggesting that demethylating agents could reactivate tumor suppressors to combat cancer progression. It should also be noted that some studies have identified an oncogenic role for miRNAs targeting TIAM1 [404,405], thus more work is needed to uncover the context-dependent effects of TIAM1 on cancer progression.

Outside of the hematopoietic system, VAV2 is critical for regulating tumorigenesis and cancer progression. As with the other central Dbl-like RhoGEFs described above, miRNAs target and regulate the expression of VAV2, leading to the suppression of cancer cell growth, migration and invasion. The study by Bischoff and colleagues found that miR-149 functioned as a key metastatic suppressor through VAV2-Rac downregulation in basal-like breast cancer [407]. Compared to luminal and HER2-positive breast cancer, the basal-like cells displayed significantly lower levels of miR-149. This group found that although the stable expression of miR-149 did not affect cell growth, it had detrimental effects on cell invasion in a Transwell assay towards multiple stimuli, including collagen and a combination of serum and EGF. A further analysis of single cells found that the distance and velocity were unchanged, but that the net displacement was reduced by miR-149, suggesting a defect in the directional migration of these cells. Mechanistically, the downregulation of VAV2 by miR-149 resulted in the impaired activation of Src and paxillin, key signaling molecules downstream of integrin engagement, drastically reducing cell spreading and the adhesion needed for migration [407]. In addition to regulating migration and invasion [406,407], the reduction of VAV2 has been linked to the inhibition of epithelial-mesenchymal transition (EMT), a process where a normally polar, epithelial cell undergoes a change to a mesenchymal-like cell to take on the characteristics of a mesenchymal cell and become more motile and invasive, as well as angiogenesis [408,409].

Similar to the Dbl family, current work suggested that DOCK family RhoGEFs typically act as oncogenic drivers of cancer. Many studies on the DOCK family have centered around their role in EMT. For example, it was shown that DOCK1 was able to promote an EMT phenotype in glioma and breast cancer [412,413]. This was shown to be accomplished through a DOCK1-mediated interleukin-8- or -22-driven NFĸB/Snail signaling mechanism. The expression of miR-31 or -486 was able to blunt this signaling pathway, promoting a non-migratory epithelial-like phenotype in cells and inhibited metastasis, through the downregulation of DOCK1 [412,413]. Similarly, the cooperation between Twist1 and BMI1 to suppress let-7i expression promoted the acquisition of properties of the EMT, namely cell migration and stem-like properties in head and neck squamous cell carcinoma (HNSCC) [414]. The loss of let-7i leads to an increase in DOCK3 expression, which promotes RAC1 activation and EMT properties. Interestingly, the re-expression of let-7i did not change the expression of EMT markers in 2D or 3D cultures, but did blunt the mesenchymal movement, invasion and metastasis [414], suggesting that let-7i can target characteristics of the EMT without directly affecting the expression of typical EMT markers.

### 5.3. MiRNA Targeting of RhoGDIs

The expression of RhoGDIs is dysregulated in many cancers and they have been shown to mediate processes directly linked to tumorigenesis and cancer progression. Much of the current work has focused on the most prominent member of the RhoGDI family, RhoGDIα. In patients with glioma, Lin et al. found that the expression of RhoGDIα protein, but not the mRNA, was frequently downregulated in the high grade glioma compared to the lower grade and matched normal brain tissue, suggesting miRNA regulation [288]. Indeed, miR-151, as well as miR-16, were found to directly target RhoGDIα mRNA to positively regulate cell migration and invasion. Interestingly, this group found that the binding of PCBP2 (multifunctional nucleic acid binding protein 2) facilitated miR-151 and -16 binding to the 3′UTR of RhoGDIα by changing the mRNA secondary structure [288], suggesting a more complex mechanism for the RhoGDIα regulation. Similar results were found in hepatocellular carcinoma [421], prostate [422] and ovarian [423] cancer, where miR-151 promoted tumor progression through processes involving synergistic cooperation with focal adhesion kinase (FAK) [421] and the reduction of Akt/mTOR signaling [423] by the direct regulation of RhoGDIα. Like the other RhoGTPase regulators, the effects of the suppression of RhoGDIα on cancer progression seem to be context dependent as the miRNA suppression of RhoGDIα in colorectal cancer resulted in suppressed cancer cell migration, invasion and metastasis [418,419].

## 6. Preclinically and Clinically Targeting miRNAs and RhoGAPs, RhoGEFs, and RhoGDIs

One question that remains to be answered is how to successfully resolve the context-dependent pro- and anti-metastatic functions of individual Rho GTPases in order to develop proper therapies. While the majority of RhoA, Rac1 and Cdc42 in vitro studies supported a pro-metastatic function, it was within in vivo studies that opposing functions were found. This may be explained by the differences in the model systems used for these experiments. Firstly, cell lines rely on key signaling pathways, including the Rho GTPases, to facilitate their adaption to rapid growth on plastic. Therefore, cell lines could be hypersensitive to any change in Rho GTPase signaling pathways not seen in in vivo systems. Additionally, it has been shown that the RhoGEF P-Rex1 was positively correlated with estrogen receptor expression and inversely correlated with PI3K levels in breast cancer [429], suggesting that the hormone receptor status and PI3K status may relate to, and be important for interpreting, the contradicting results in vitro. Secondly, Rho GTPases may also be involved in positive or negative feedback loops with the tumor microenvironment that regulate the metastatic potential of a tumor, which is not completely mimicked in the cell culture systems. Lastly, manipulating the expression levels of single Rho GTPase in cell lines may disrupt the level or activity of other Rho GTPases within the cell, affect homeostasis and lead to a poor drug efficacy.

Rho GTPases themselves are not considered viable clinical targets. This is due to their (1) structure, which provides limited small-molecule binding pockets, (2) high affinity for GTP and GDP, and (3) high concentration of GTP available in the cells [430,431]. Therefore, a more reasonable approach to limit Rho GTPases in cancer is to target the activators of Rho GTPases. Since the enhanced activation or overexpression of Rho GTPases is common in cancer [432,433], these treatment strategies primarily revolve around blocking the interaction of Rho GTPases with GEFs or GTP nucleotide binding. By targeting GEFs or GTP binding, Rho GTPases lose the ability to quickly exchange GDP for GTP leading to the inhibition of the interaction with downstream effectors, which thus inhibits signaling cascades. Both targeting strategies have been shown to be effective for cancer and have been reviewed more extensively, along with other Rho GTPase targeting strategies, which can be found elsewhere [430,431,434,435].

However, a more straightforward approach to regulating Rho GTPases is to manipulate miRNA expression. As discussed above, a growing body of evidence has demonstrated that miRNAs are effective at regulating Rho GTPase activity in cancer by targeting RhoGAPs, RhoGEFs and RhoGDIs. This provides a rationale for manipulating the miRNAs as a treatment strategy. The main advantage of using miRNAs as therapeutics is that a single miRNA can affect multiple pathways, avoiding the “one-drug, one-target” approach that often leads to resistance [436]. The use of the so-called miRNA mimics is the main and most effective approach for restoring or replacing tumor-suppressing miRNA expression. These are often chemically modified RNA duplexes that can be loaded into the RISC and act similarly to a mature miRNA on downstream targets. Many studies have shown the efficacy of this approach to regulate miRNA targets such as the Rho GTPases and Rho GTPase regulators [361,437,438,439]. These studies have highlighted the potential utility of miRNAs in the clinic, and provided supporting evidence for ongoing miRNA mimics in clinical trials (miR-16 (Mesomir 1), miR-34 (MRX34), and miR-29b (MRG-201)). However, miRNA mimics have hit many roadblocks that currently limit its use in clinics.

On the other hand, the development of miRNA inhibitors to target oncogenic miRNAs is a burgeoning area of research for clinical use. This includes the development of small molecule inhibitors of miRNAs (SMIRs), locked nucleic acid (LNA) antimiRs, antagomiRs and miRNA sponges. Gumireddy and colleagues reported the first SMIR in 2008, when they found a small molecule that effectively inhibited the transcription of miR-21 [440]. Since then, other SMIRs have been identified [441,442,443], however none of these SMIRs have yet been shown to result in an altered expression of the Rho GTPase regulators. LNAs are bicyclic RNA analogs that contains a 2′-O, 4′-C methylene bridge that restricts the flexibility of the ring [444]. LNAs possess an extremely high affinity and specificity for complementary DNA and RNA sequences, making them effective for antagonizing miRNA function [445]. LNAs have been successfully employed resulting in increased levels of RhoGAPs in culture [301]. AntagomiRs are single-stranded RNA molecules designed to be complementary to the target miRNA [446] and have been shown to effectively regulate the activity of Rho GTPases in vivo [438,447]. The last method for miRNA inhibition involved miRNA sponges, or competitive inhibitors that contained multiple, tandem seed sequence binding sites to a miRNA of interest and could inhibit all miRNAs that shared the seed sequence that it expressed [448]. MiRNA sponges have been used to enforce the expression of Rho GTPase regulators in the brain and liver [449,450]. Each of these strategies effectively sequestered the target miRNA and prevented it from being loaded into the RISC. This resulted in the enhanced expression of the miRNA target. However, as with the miRNA mimics, inhibitors of miRNAs are still early in terms of clinical trial effectiveness, but show promise.

Although the targeting of Rho GTPase regulators for cancer with miRNA replacement or miRNA inhibitors is a straightforward idea, the two biggest barriers that miRNA interventions face are the stability issue and the development of effective delivery systems. RNA molecules are inherently unstable due to their 2′-OH chemical group [451,452]. However, recent advances in our understanding of non-coding RNA (ncRNA) biology has led to the production of natural or chemical modifications that increase RNA stability, seen in many of the miRNA inhibitors discussed above. In contrast, the effective delivery of miRNA remains the biggest challenge for miRNA therapies. Delivery systems are currently either viral- or non-viral-based. The toxicity and immunogenicity related to viral vectors limit their use in the clinic, therefore non-viral-based delivery systems have continued to evolve as a promising approach. In addition to the lack of toxicity and immunogenicity, the tolerance of the cargo size and the ease of control over the composition, modification and manufacturing add to the attraction of non-viral-based delivery systems for ncRNA therapies [453,454,455]. In particular, cationic materials that condense negatively charged nucleic acids through electrostatic interactions have shown the efficiency and specificity needed for clinical miRNA therapies. Recent work has tried to modify particle size and surface composition to increase the lower efficiency of delivery when compared to viral vectors and avoid unexpected biological outcomes [453,456,457,458].

## 7. Conclusions

**Figure 3:**
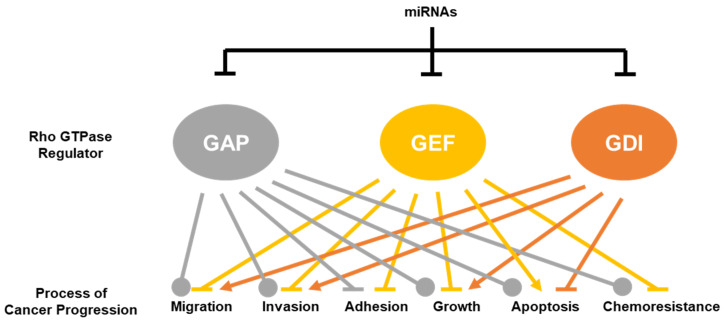
miRNA targeting of the Rho GTPase regulators has distinct effects on the processes of cancer progression. The downregulation of small Rho GTPase regulators (RhoGAPs, RhoGEFs, and RhoGDIs) has different effects on the processes known to drive the cancer progression. Much of the literature agrees that the downregulation of RhoGEFs results in the inhibition of progression, whereas the downregulation of RhoGDIs promotes cancer progression. Currently, there are conflicting results on the effects of RhoGAPs downregulation on cancer progression, with about half demonstrating inhibition and the other half promoting progression. Arrowheads (→) signify a positive effect on cancer progression. (┴) signifies a negative effect. (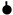) signifies that both positive and negative effects have been frequently reported in the literature.

This review clearly demonstrated that miRNAs are involved in regulating multiple facets of cancer cell biology through the regulation of RhoGAPs, RhoGEFs and RhoGDIs. A comprehensive list of the currently validated RhoGAP, RhoGEF, and RhoGDI miRNA targets is shown in Table 3. Although the targeting of RhoGAPs results in much more context-dependent effects on cancer progression, the current data generally suggest that miRNAs that target RhoGEFs and RhoGDIs act as tumor suppressors and oncogenes, respectively (Figure 3).

The data summarized here demonstrate a strong rationale for targeting Rho GTPase regulators as a potential therapeutic approach for aggressive and highly metastatic cancers. However, no clinically effective drugs targeting these regulators have been approved for cancer therapy. Current studies revolve around inhibiting activators of Rho GTPases (RhoGEFs), however compounds that mimic RhoGAPs by inserting an arginine residue or stimulate GTP hydrolysis may provide another promising avenue for treatment.

MiRNAs are proving to be advantageous in the clinic, particularly in diseases such as cancer, that may not have a single underlying cause. Moreover, some small molecule drugs are currently being developed to target multiple pathways [459], lending credence to the use of miRNAs clinically. In order to better utilize miRNAs that therapeutically target the Rho GTPase regulators, future work will need to better identify the context-dependent effects and mechanisms of the miRNA targeting of RhoGAPs, RhoGEFs and RhoGDIs. Moreover, identifying what leads to the context-dependent tumor suppressor or oncogenic effects of a single miRNA in different cancers will be critical not only to our basic knowledge of cancer progression, but also to enhance the clinical utility for that miRNA (for example, miR-141 targets ARHGAP7 (DLC1) to promote colorectal cancer cell growth, migration and invasion [306], but inhibits liver cancer progression by targeting TIAM1 [400]). In terms of the regulators themselves, future work will need to address whether GEMs directly modulate Rho GTPase activity and signaling as well as the specificity of GEMs for certain members of the Rho GTPase family. Accomplishing both will not only advance our understanding of the functions of these regulators in cancer, but it will also identify other potential miRNA and Rho GTPase regulator targets, increasing their appeal as therapeutics. Additionally, although significant strides have been taken to increase the stability and delivery systems for miRNAs, more work needs to be done to improve the targeting ability of these systems and to enhance therapeutic effects without triggering an immune system response. Even though the use of miRNAs in a clinical setting is currently limited, the recent FDA approval of siRNA for the treatment of the peripheral nerve disease caused by hereditary transthyretin-mediated amyloidosis is promising for ncRNA therapy [460]. Overall, the wide range of interactions between miRNAs and RhoGAPs, RhoGEFs and RhoGDIs in various cancers provides new challenges and opportunities for the development of new general and personalized therapeutic strategies.

## Figures and Tables

**Table 1:** Mammalian Rho GTPases.

GTPase (Alias)	Function	Citation
CDC42 (CDC42Hs)	Transduced signals to the actin cytoskeleton to initiate and maintain polarized growth and mitogen-activate protein morphogenesis; Responsible for the formation of filopodia in actin-based cell migration.	[32,33]
RAC1	Transduced signals to the actin cytoskeleton to regulate the multiple signaling pathways that control actin cytoskeleton organization, transcription and proliferation; Responsible for the formation of lamellipodia in actin-based cell migration.	[34]
RAC2	Transducer that localized to the plasma membrane, where it worked with Rac1 to regulate diverse processes, such as secretion, phagocytosis and cell polarization; Expressed primarily in hematopoietic cell lineages.	[35,36]
RAC3	Involved in synaptic potentiation through regulating the actin cytoskeletal dynamics; Primarily expressed in the neurons of ganglia and the central nervous system.	[37,38]
RHOA	Involved in the regulation of cell adhesion and migration; Responsible for providing contractile force in cell migration through the formation of stress fibers and focal adhesions; Localized to the cytoplasm and to a certain degree the plasma membrane.	[39,40]
RHOB	Transducer involved in actin organization, cell migration, membrane and endosome trafficking, proliferation, DNA repair, and apoptosis; Thought to be an inhibitor of cancer progression; Localized to the endosomal membrane.	[28,41]
RHOBTB1	Function not well known, did not play a major role in the organization of actin cytoskeleton dynamics; Not targetable by RhoGAPs, RhoGEFs, or RhoGDIs; Ubiquitously expressed, although high levels are found in skeletal muscle, placenta, stomach, kidney, testis, adrenal gland and uterus.	[42,43]
RHOBTB2	Function not well known, did not play a major role in the organization of actin cytoskeleton dynamics; Not targetable by RhoGAPs, RhoGEFs, or RhoGDIs; Weakly expressed, although high levels were found in neural and cardiac tissues.	[42,43]
RHOBTB3	Function not well known, did not play a major role in the organization of actin cytoskeleton dynamics; Not targetable by RhoGAPs, RhoGEFs, or RhoGDIs; Ubiquitously expressed, although high levels were found in placenta, testis, pancreas, adrenal and salivary gland, and neural and cardiac tissues.	[42,43]
RHOC	Responsible for actin cytoskeletal reorganization involved in promoting cell migration, proliferation, EMT, invasion, angiogenesis and metastasis; Localized to the cytoplasm and with undefined perinuclear structures.	[28,44]
RHOD	Controlled endocytic vesicle movement, Golgi homeostasis, and promoted actin stress fiber dissociation; Localized to the endosomal membrane.	[28,45]
RHOE (RND3)	Expressed ubiquitously; Inhibited contractility and the subsequent formation of actin stress fibers and focal adhesions; Drove cell rounding. GTPase-deficient, but constitutively bound to GTP in vivo.	[28]
RHOF (RIF)	Expressed ubiquitously; Promoted the formation of filopodia.	[28]
RHOG	Localized to caveolar vesicles; May have played a role in the inflammatory response; Involved in lamellipodia and filopodia formation, and membrane ruffling.	[28,38]
RHOH (TTF)	Expressed primarily in hematopoietic cell lineages; GTPase-deficient; Overexpression inhibited RAC1, RHOA, and CDC42 signaling; Not targetable by RhoGAPs, RhoGEFs, or RhoGDIs.	[28,36]
RHOJ (TCL)	Localized to the endosomal membrane; Promoted the formation of filopodia; Contributed to the focal adhesion turnover.	[1,28]
RHON (RND2)	Expressed primarily in testis, brain, and liver; GTPase-deficient; Involved in neurite outgrowth and cytokinesis.	[28,46]
RHOQ (TC10)	Localized to the endosomal membrane; Promoted the formation of filopodia; Implicated in receptor trafficking.	[28,46]
RHOS (RND1)	Expressed primarily in adult brain and liver; Inhibited contractility and the subsequent formation of actin stress fibers and focal adhesions; Drove cell rounding; GTPase-deficient.	[28,46]
RHOU (WRCH1)	Critical for Wnt signaling; Worked together with RAC1; Stimulated cell cycle progression; Promoted dissociation of stress fibers.	[1,28]
RHOV (WRCH2)	Promoted the formation of filopodia; Promoted the dissociation of stress fibers.	[28,46]

RhoGAPs: Rho GTPase-activating proteins, RhoGEFs: Rho guanine nucleotide exchange factors, RhoGDIs: Rho guanosine diphosphate (GDP) dissociation inhibitors (GDIs).

**Table 2:** Mammalian Rho GTPase regulators and their specificity for Rho GTPases in mammals.

Function	Gene	Aliases	Rho GTPase Specificity	Citation
GTPase-activating protein (GAP)	ABR ^@^	MDB	RHOA, RAC1, RAC2, RAC3, CDC42	[51,52]
	ARAP1	CENTD2	RHOA, CDC42	[53]
	ARAP2	CENTD1, PARX	RHOA *	[54]
	ARAP3	CENTD3, DRAG1	RHOA, RAC1, CDC42	[55,56]
	ARHGAP1	CDC42GAP, p50RHOGAP, RHOGAP, RHOGAP1	RHOA, RHOB, RHOC, RHOG, RHOJ, RHOQ, RAC1, RAC2, RAC3, RHOG, CDC42	[51]
	ARHGAP2	CHN1, n-CHIMERIN, DURS2, RHOGAP2	RAC1, CDC42	[57]
	ARHGAP3	CHN2, β-CHIMERIN, RHOGAP3	RAC1, CDC42	[51]
	ARHGAP4	SrGAP4, p115RHOGAP, RHOGAP4	RHOA, RAC1, CDC42	[58,59,60]
	ARHGAP5	GFI2, p190BRHOGAP, RHOGAP5	RHOA, RAC1, CDC42	[61]
	ARHGAP6	RHOGAPX-1, RHOGAP6	RHOA, RAC3	[62,63]
	ARHGAP7	DLC1, STARD12, p122RHOGAP	RHOA, RHOB, RHOC, CDC42	[51,64]
	ARHGAP8	PP610, BPGAP1	RHOA, RAC1, CDC42	[65]
	ARHGAP9	10C, RGL1, MGC1295	RHOA, RAC1, CDC42	[66]
	ARHGAP10	GRAF2, PSGAP	RHOA, CDC42	[67]
	ARHGAP11A	MPGAP, GAP(1-12)	RHOA	[68]
	ARHGAP11B	B’-T, FAM7B1, GAP(1-8)	Not yet determined (Not RHOA)	[69]
	ARHGAP12		RAC1, CDC42	[70]
	ARHGAP13	SRGAP1, NMTC2	RHOA, CDC42	[71]
	ARHGAP14	SRGAP3, WRP, MEGAP	RAC1, CDC42	[72,73]
	ARHGAP15	BM046	RAC1	[51]
	ARHGAP16P		Not yet determined	
	ARHGAP17	RICH1, NADRIN, WBP15	RHOA, RHOQ, RAC1, RAC2, CDC42	[51,74]
	ARHGAP18	MACGAP, SENEX	RHOA, RHOC	[75,76]
	ARHGAP19		RHOA	[77]
	ARHGAP20	RARHOGAP	RHOA, RAC1, CDC42	[78]
	ARHGAP21		RHOA, RHOC, CDC42	[48,79]
	ARHGAP22	RHOGAP22	RAC1	[80]
	ARHGAP23		Not yet determined	
	ARHGAP24	FILGAP, RCGAP72, p73RHOGAP	RAC1, CDC42	[81,82]
	ARHGAP25	HEL-S-308	RAC1, CDC42	[83]
	ARHGAP26	GRAF1, OPHN1L	RHOA, RHOB, RHOC, RHOJ, RHOQ, RAC1, CDC42	[51]
	ARHGAP27	CAMGAP1, SH3D20	RAC1, CDC42	[82,84]
	ARHGAP28		RHOA	[85]
	ARHGAP29	PARG1	RHOA, RAC1, CDC42	[86]
	ARHGAP30		RHOA, RAC1	[87]
	ARHGAP31	CDGAP, AOS1	RAC1, CDC42	[88]
	ARHGAP32	GCGAP, GRIT, RICS, p200RHOGAP	RHOA, RAC1, CDC42	[89]
	ARHGAP33	TCGAP, NOMAGAP, SNX26	RHOA, RHOQ, RAC1, CDC42	[90]
	ARHGAP34	SRGAP2, FNBP2	RAC1	[91,92,93]
	ARHGAP35	GRLF1, p190RHOGAP	RHOA, RHOB, RHOC, RHOD, RHOJ, RHOQ, RAC1, RAC2, RAC3, CDC42	[51]
	ARHGAP36		RAC1 *^,$^, CDC42 *^,$^	[94]
	ARHGAP37	STARD13, DLC2, GT650	RHOA, CDC42	[95,96]
	ARHGAP38	STARD8, DLC3, STARTGAP3	RHOA, CDC42	[97]
	ARHGAP39	CRGAP, VILSE	RAC1, CDC42	[98]
	ARHGAP40		Not yet determined	
	ARHGAP41	OPHN1, OPN1, MRX60	RHOA, RHOB, RHOC, RAC1, RAC2, RAC3, CDC42, RHOQ, RHOJ	[51]
	ARHGAP42	TMEM133, GRAF3, AD031	RHOA	[99]
	ARHGAP43	SH3BP1	RAC1, CDC42	[100,101]
	ARHGAP44	RICH2, NPC-A-10	RAC1, CDC42	[102,103]
	ARHGAP45	HMHA1, HA-1, HLA-HA1	RHOA, RAC1	[104]
	ARHGAP46	GMIP	RHOA	[105]
	ARHGAP47	TAGAP, FKSG15, IDDM21	RHOA, RHOH *	[106,107]
	ARHGAP48	FAM13A	RHOA	[108]
	ARHGAP49	FAM13B	Not yet determined	
	BCR ^@^	ALL, CML, PHL	RHOA, RAC1, RAC2, CDC42	[51,52]
	INPP5B	5PTase	RAC1, CDC42	[109]
	INPP5F	OCRL, LOCR, DENT-2, NPHL2	RHOA, RAC1, CDC42	[110,111]
	IQGAP1	SAR1, HUMORFA01, p195	RHOA *, RHOB *, RAC1 *, CDC42 *	[112,113,114,115]
	IQGAP2		RHOC *, RAC1 *, CDC42 *	[116,117]
	IQGAP3		RAC1 *, CDC42 *	[118]
	MYO9B	MYR5, CELIAC4	RHOA, RAC1, CDC42	[119]
	PIK3R1	p85ALPHA, p85α, AGM7, GRB1, IMD36	RAC1, CDC42	[120,121]
	PIK3R2	p85BETA, p85β, MPPH	RAC1, CDC42	[122]
	RACGAP1	MGCRACGAP, CYK4, IDGAP	RHOA, RHOD, RAC1, RAC2, RAC3, CDC42	[51,123,124]
	RALBP1	RIP1, RLIP1, RLIP76	RAC1, CDC42	[125]
Guanine nucleotide exchange factor (GEF)	ALS2	ALS2CR6, ALSJ, IAHSP, PLSJ	RAC1	[126]
	ALS2CL	RN49018	Not yet determined (Not RHOA, RAC1, or CDC42)	[127]
	ARHGEF1	IMD62, LBCL2, LSC, p115RHOGEF	RHOA, RHOB, RHOC, RAC1^$^, CDC42^$^	[94,128,129]
	ARHGEF2	GEFH1, LFP40, LFC, GEF2	RHOA, RHOB, RAC1	[130,131,132]
	ARHGEF3	XPLN, GEF3, STA3	RHOA, RHOB	[133]
	ARHGEF4	ASEF, GEF4, SMIM39, STM6	RAC1, CDC42	[134,135]
	ARHGEF5	GEF5, TIM1	RHOA, RHOB, RHOC, RHOG, RAC1, CDC42	[136,137,138]
	ARHGEF6	COOL2, MRX46, PIXA, ALPHAPIX	RAC1, CDC42	[139]
	ARHGEF7	BETAPIX, COOL1, PIXB	RAC1, CDC42	[139]
	ARHGEF8	NET1	RHOA, RHOB	[140,141]
	ARHGEF9	HPEM-2, PEM2	CDC42	[142]
	ARHGEF10	GEF10, SNCV	RHOA, RHOB, RHOC	[143]
	ARHGEF10L	GRINCHGEF	RHOA, RHOB, RHOC	[144]
	ARHGEF11	GTRAP48, PDZRHOGEF	RHOA, RHOB, RHOC	[129,145]
	ARHGEF12	LARG, PRO2792	RHOA, RHOB, RHOC	[129,146]
	ARHGEF13	AKAP13, BRX, LBC, PRKA13	RHOA, RHOB, RHOC	[147]
	ARHGEF14	MCF2L, DBS, OST	RHOA, CDC42	[148]
	ARHGEF15	EPHEXIN5, VSMRHOGEF	RHOA, RAC1, CDC42	[149,150,151]
	ARHGEF16	GEF16, NBR	RHOG, RAC1 ^#^, CDC42	[152,153,154]
	ARHGEF17	RHOGEF17, TEM4, p164RHOGEF	RHOA, RHOB, RHOC	[155,156,157]
	ARHGEF18	RP78, SARHOGEF, p114RHOGEF	RHOA, RAC1	[158,159]
	ARHGEF19	WGEF	RHOA, RAC1, CDC42	[160]
	ARHGEF21	MCF2, DBL	RHOA, RHOB, RHOC, RHOG, CDC42	[161,162]
	ARHGEF22	MCF2L2	RHOA, RAC1, CDC42	[163,164]
	ARHGEF23	TRIO, MEBAS, MRD44	RHOA, RHOG, RAC1	[165,166]
	ARHGEF24	KALRN, DUO, CDH5, TRAD, HAPIP	RHOA ^#^, RHOB ^#^, RHOC, RHOG, RAC1, RAC2, CDC42 ^#^	[167,168,169]
	ARHGEF25	GEFT, p63RHOGEF	RHOA, RAC1, CDC42	[170,171,172]
	ARHGEF26	SGEF, CSGEF, HMFN1864	RHOG	[173]
	ARHGEF27	NGEF, EPHEXIN	RHOA, RAC1, CDC42	[174]
	ARHGEF28	RGNEF, RIP2, p190RHOGEF	RHOA, RHOB, RHOC, RAC1^$^	[129,146,175]
	ARHGEF29	SPATA13, ASEF2	RHOA, RAC1, CDC42	[176,177]
	ARHGEF30	OBSCN, UNC89	RHOA, RHOQ	[178,179]
	ARHGEF31	ECT2	RHOA, RHOB, RHOC, RHOG, RAC1, CDC42	[141,180,181,182,183]
	ARHGEF32	ECT2L, LFDH, FBXO49	Not yet determined	
	ARHGEF33		Not yet determined	
	ARHGEF34P		Not yet determined	
	ARHGEF35	ARHGEF5L	Not yet determined	
	ARHGEF36	DNMBP, TUBA, CTRCT48	CDC42	[184]
	ARHGEF37	TUBA3	CDC42^$^	[184]
	ARHGEF38	TUBA2	CDC42^$^	[184]
	ARHGEF39		RAC1	[185]
	ARHGEF40	SOLO	RHOA, RHOC, RAC1, RAC2, RAC3, CDC42	[186,187]
	ARHGEF41	PLEKHG1	RAC1, CDC42	[188]
	ARHGEF42	PLEKHG2, CLG, LDAMD	RAC1, CDC42	[189]
	ARHGEF43	PLEKHG3	RAC1, CDC42	[190]
	ARHGEF44	PLEKHG4, SCA4, PRTPHN1	RHOA, RAC1, CDC42	[191]
	DEF6	IBP, SLAT, SWAP70L	RHOA, RAC1, CDC42	[192,193]
	FARP1	CDEP, PLEKHC2	RHOA, RAC1, CDC42	[94,194,195]
	FARP2	FIR, FRG, PLEKHC3	RAC1, CDC42	[196,197]
	FGD1	AAS, FGDY, MRXS16, ZFYVE3	CDC42	[198]
	FGD2	ZFYVE4	CDC42	[199]
	FGD3	ZFYVE5	CDC42	[200]
	FGD4	CMT4H, FRABP, ZFYVE6	CDC42	[201]
	FGD5	ZFYVE23	RAC1, CDC42	[202,203]
	FGD6	ZFYVE24	CDC42	[204]
	ITSN1	ITSN, SH3D1A, SH3P17	CDC42	[205]
	ITSN2	PRO2015, SH3D1B, SH3P18, SWA, SWAP	CDC42	[206]
	PLD2	PLD1C	RHOA, RAC2	[207,208]
	PLEKHG4B		Not yet determined	
	PLEKHG5	CMTRIC, DSMA4, GEF720, SYX, TECH	RHOA	[209]
	PLEKHG6	MYOGEF	RHOA, RHOC, RHOG, RAC1	[210,211,212]
	PLEKHG7		Not yet determined	
	PREX1	P-REX1	RHOA, RAC1, RAC2, CDC42	[213]
	PREX2	DEPDC2, P-REX2	RHOG, RHOQ, RAC1, RAC2, RAC3, CDC42	[214,215,216]
	RASGRF1	GNRP, GRF1, CDC25, GRF55, CDC25L	RAC1, CDC42	[217,218]
	RASGRF2	GRF2	RAC1, CDC42	[218,219]
	RAP1GDS1	GDS1, SMGGDS	RHOA, RHOC, CDC42	[220,221]
	SOS1	GF1, GGF1, GINGF, HGF, NS4	RHOA, RAC1	[222,223]
	SOS2	NS9	RAC1	[222]
	SWAP70	HSPC321	RAC1, RAC2	[224,225]
	TIAM1		RHOA, RAC1, CDC42	[226]
	TIAM2	STEF	RAC1, CDC42^$^	[227,228,229]
	VAV1	VAV	RHOA, RHOG, RAC1, CDC42	[230,231,232]
	VAV2		RHOA, RHOB, RHOG, RAC1, CDC42	[230,231,232,233]
	VAV3		RHOA, RHOG, RAC1, CDC42	[230,233,234]
	DOCK1	DOCK180, CED5	RAC1, RAC2, RAC3	[235,236,237]
	DOCK2	IMD40	RHOA, RAC1, RAC2	[223,238,239]
	DOCK3	MOCA, NEDIDHA, PBP	RAC1	[240]
	DOCK4		RAC1	[241]
	DOCK5		RAC1, RAC2^$^, RAC3^$^	[237,242]
	DOCK6	AOS2, ZIR1	RAC1, CDC42	[243]
	DOCK7	EIEE23, ZIR2	RAC1, RAC3, CDC42	[244,245]
	DOCK8	HEL-205, MRD2, ZIR8	CDC42	[246]
	DOCK9	ZIZ1, ZIZIMIN1	CDC42	[247]
	DOCK10	DRIP2, ZIZ3	RHOJ^$^, RHOQ^$^, CDC42	[248,249]
	DOCK11	ACG, ZIZ2	CDC42	[249]
GDP-dissociation inhibitor (GDI)	ARHGDIA	GDIA1, HEL-S-47e, NPHS8, RHOGDI	RHOA, RHOB^$^, RHOC, RHOG, RAC1, RAC2, CDC42	[250,251,252,253,254]
	ARHGDIB	D4, GDIA2, LYGDI, RAP1GN1, RHOGDI2	RHOA, RAC1, CDC42	[251,255,256]
	ARHGDIG	RHOGDI3	RHOA, RHOB, RHOG, CDC42	[257,258,259]

* binds Rho GTPase, but does not stimulate the hydrolysis of GTP. # binds Rho GTPase, but does not facilitate the exchange of GDP for GTP. @ functions both as a GAP and a GEF. $ binding found, but no GTPase activity was analyzed; ABR: Active breakpoint cluster region-related protein, ALS2: Alsin rho guanine nucleotide exchange factor 2, ALS2CL: ALS2 C-terminal like, ARAP: ArfGAP with RhoGAP domain, ankyrin repeat and PH domain, ARHGAP: Rho GTPase activating protein, ARHGAP16P: Rho GTPase activating protein 16 pseudogene, ARHGEF: Rho guanine nucleotide exchange factor, ARHGEF10L: Rho guanine nucleotide exchange factor 10 like, ARHGDI: Rho GDP dissociation inhibitor, BCR: Breakpoint cluster region protein, DEF6: Differentially expressed in FDCP 6, DOCK: Dedicator of cytokinesis, FARP: FERM, ARH/RhoGEF and pleckstrin domain protein, FGD: FYVE, RhoGEF and PH domain containing, INPP: Inositol polyphosphate-1-phosphatase, IQGAP: IQ motif containing GTPase activating protein, ITSN: Intersectin, MYO9B: Myosin IXB, PIK3R: Phosphoinositide-3-kinase regulatory subunit, PLD2: Phospholipase D2, PLEKH: Pleckstrin homology and RhoGEF domain containing, PREX: Phosphatidylinositol-3,4,5-triphosphate dependent Rac exchange factor, RACGAP: Rac GTPase activating protein, RALBP: RalA binding protein, RAP1GDS1: Rap1 GTPase-GDP dissociation stimulator 1, RASGRF: Ras protein specific guanine nucleotide releasing factor, SOS: Son of sevenless, SWAP70: Switch associated protein 70, TIAM: T-lymphoma invasion and metastasis-inducing protein, VAV: Vav guanine nucleotide exchange factor.

**Table 3:** RhoGAPs, RhoGEFs and RhoGDIs targeted by microRNAs in cancer.

Rho GTPase Regulator	Direct Target of the miRNA	miRNA	miRNA is a Tumor Suppressor or Oncogene	Effect of microRNA Targeting	Reference
Rho GAPs	ABR	miR-762	oncogene	Enhanced survival; Promoted gefitinib resistance; Promoted tumor formation.	[294]
	ARHGAP1	miR-19a	n/a	No effects on cells were assayed in this publication for ARHGAP1.	[295]
	ARHGAP1	miR-34a/ miR-509	tumor suppressor	Inhibited TGF-B-induced tumor cell invasion and metastasis; Inhibited growth, invasion and migration; Sensitized cells to cisplatin.	[296,297]
	ARHGAP1	miR-130b/ miR-940	oncogene	Enhanced CDC42 activity which led to enhanced AP-1-mediated growth, migration and invasion; Induced extensive osteoblastic lesions in calvarian tumors; drove osteogenic differentiation in mesenchymal stem cells.	[298,299]
	ARHGAP5	miR-486	tumor suppressor	Inhibited migration, invasion and metastasis.	[300]
	ARHGAP5	miR-494	oncogene	Enhanced the invasion of glioma cells through increased EGFR stabilization and the subsequent activation of ERK and Akt.	[301]
	ARHGAP5	miR-744	oncogene	Promoted cell migration and invasion; Directly interacted with the ARHGAP5 promoter to reduce expression.	[302,303]
	ARHGAP7	miR-18a/ miR-106b/ miR-141/ miR-301a/ miR-429/ miR-483	oncogene	Promoted growth, migration, invasion and EMT; Reduced cells in the G0/G1 phase of the cell cycle; Regulated by IGF2.	[304,305,306,307,308,309]
	ARHGAP7	miR-200a/ miR-200b/ miR-200c	tumor suppressor	Reversed EMT and inhibited lung metastatic gene expression; Enhanced proliferation and colony formation, but reduced migration and invasion.	[310,311]
	ARHGAP9	miR-224	oncogene	Drove cell migration and invasion by the activation of upstream LPS, LTα, and TNFα inflammatory pathways.	[312]
	ARHGAP10	miR-337	tumor suppressor	Reduced migration, invasion and viability, but no effects on cell cycle.	[313]
	ARHGAP10	miR-3174	oncogene	Inhibited apoptosis and autophagy; Contributed to cisplatin resistance.	[314]
	ARHGAP12	miR-20a	n/a	No effects on cells were assayed in this publication for ARHGAP12.	[315]
	ARHGAP13	miR-124/ miR-340	tumor suppressor	Negatively modulated ROCK1, MET and CTGF; Reduced proliferation, colony formation, migration and invasion; Promoted G1 cell cycle arrest and decreased cells in S-phase; Decreased phosphorylation of Rb.	[316]
	ARHGAP13	miR-145	oncogene	Drove an invasive phenotype.	[317]
	ARHGAP18	miR-153/ miR-200b	tumor suppressor	Sponged (ceRNA) by CDKN2BAS to drive metastasis; Inhibited cell growth, migration, invasion, and metastasis; Enhanced stress fiber formation.	[50,318]
	ARHGAP19	miR-200c	tumor suppressor	Suppressed anoikis resistance, migration, and EMT.	[319]
	ARHGAP21	miR-224	oncogene	Drove cell migration and invasion by the activation of upstream LPS, LTα and TNFα inflammatory pathways.	[312]
	ARHGAP24	miR-590	oncogene	Promoted cell viability, migration and invasion; Inhibited apoptosis.	[320]
	ARHGAP26	miR-30b/ miR-573	n/a	No effects on cells were assayed in this publication for ARHGAP26.	[321]
	ARHGAP29	miR-200b	tumor suppressor	Inhibited migration and invasion; Drove actin cytoskeleton reorganization; Inhibited invadopodia formation.	[322]
	ARHGAP37	miR-9/ miR-125b	oncogene	Promoted PDGFRB-induced angiogenic tube formation in vitro and vascular lacunae in vivo; Increased the proliferation, migration, invasion, EMT and metastasis; Drove the expression of a-SMA and vimentin through RHOA/ROCK signaling.	[323,324,325,326]
	BCR	miR-23a/ miR-320a	tumor suppressor	Inhibited growth and EMT; Drove cellular senescence; Blunted phosphorylation of PI3K, Akt, and NF-KB.	[327,328]
	IQGAP1	miR-124	n/a	Drove gene expression profile to that of the brain.	[329]
	IQGAP1	miR-124/ miR-203/ miR-506	tumor suppressor	Inhibited cell growth, migration, invasion, and adhesion; Induced cell cycle arrest at the G1-S checkpoint; Reversed EMT; Repressed ERK activation.	[330,331,332,333]
	IQGAP2	miR-92a	n/a	No effects on cells were assayed in this publication for IQGAP2.	[334]
	PIK3R1	miR-21/ miR-155/ miR-487a	oncogene	Promoted growth, invasion, EMT and metastasis; Increased MAPK and PI3K-Akt activation; Drove gemcitabine resistance.	[335,336,337,338]
	PIK3R1	miR-29a	n/a	Inhibited Akt phosphorylation; Prevented the insulin-mediated inhibition of PEPCK.	[339]
	PIK3R1	miR-29a/ miR-200b/ miR-200c/ miR-128/ miR-218/ miR-221/ miR-376a/ miR-486/ miR-503/ miR-542	tumor suppressor	Suppressed growth, migration and invasion; Induced apoptosis; Blunted PI3K/Akt/mTOR and MMP9 signaling; Upregulated p53 expression; Enhanced chemosensitivity to gemcitabine and temozolomide.	[340,341,342,343,344,345,346,347,348]
	PIK3R2	miR-30a/ miR-126/ miR-323/ miR-608	tumor suppressor	Inhibited growth, migration and invasion; Decreased VEGF/PI3K/Akt signaling pathway activation; Suppressed G2/M cell cycle transition; Promoted EGFR-inhibitor sensitivity.	[349,350,351,352,353,354,355]
	RACGAP1	miR-192/ miR-204/ miR-4324	tumor suppressor	Inhibited growth, migration, invasion, and metastasis; Induced G0/G1 cell cycle arrest.	[356,357,358]
Rho GEFs	ARHGEF1	miR-143	tumor suppressor	Inhibited migration, invasion, tumor growth and metastasis; Lowered the activities of RHOA, RAC1, and CDC42; Increased E-cadherin protein expression.	[359]
	ARHGEF2	miR-143/ miR-194	tumor suppressor	Inhibited growth, migration, invasion, tumor growth and metastasis; Lowered the activities of RHOA, RAC1, and CDC42; Increased E-cadherin protein expression.	[359,360]
	ARHGEF3	miR-138/ miR-200b	tumor suppressor	Suppressed migration and invasion; Reorganized the stress fibers to a more rounded shape; Inhibited invadopodia formation.	[322,361]
	ARHGEF6	miR-23b/ miR-135a	tumor suppressor	Inhibited migration, invasion, spreading, adhesion, tumor growth, and metastasis; Inhibited lamellipodia formation; Blunted tumor initiation of CSCs.	[362,363]
	ARHGEF8	miR-22/ miR-24	oncogene	Increased colony formation, invasion, EMT, and chemoresistance; Decreased apoptosis.	[364,365]
	ARHGEF8	miR-22/ miR-200b/ miR-206	tumor suppressor	Decreased growth, migration, invasion, and chemoreistance; Inhibited stress fiber and invadopodia formation.	[322,366,367]
	ARHGEF19	miR-29b/ miR-503	tumor suppressor	Reduced growth, migration, invasion, tumor growth and metastasis; Blunted ERK signaling.	[368,369]
	ARHGEF25	miR-874/ miR-3189	tumor suppressor	Decreased growth, migration and invasion; Induced apoptosis.	[370,371]
	ARHGEF31	miR-194/ miR-223/	tumor suppressor	Decreased tumor growth, viability, migration, and invasion; Induced apoptosis; Induced p21, p27, and Rb expression.	[372,373,374]
	FGD1	miR-200c	oncogene	Promoted lung metastasis.	[375]
	FGD4	miR-17- 92a cluster	tumor suppressor	Decreased growth, EMT and migration; Delayed tumor onset; Reduced Akt and ERK activation.	[376]
	PLD2	miR-203/ miR-887/ miR-3619	tumor suppressor	Blunted growth, migration and invasion.	[377,378]
	PREX2	miR-338	tumor suppressor	Inhibited growth, migration and invasion; Induced G1 cell cycle arrest; Activated PTEN.	[379,380]
	RASGRF1	miR-137/ miR-709	tumor suppressor	Decreased growth, migration and invasion; Enhanced apoptosis; Reduced initiation and maintenance of leukemogenesis.	[381,382]
	SOS1	miR-20b/ miR-124/ miR-143/ miR-146a/ miR-148a/ miR-628/ miR-4728	tumor suppressor	Inhibited growth, viability, migration, invasion and EMT; Promoted apoptosis; Reduced ERK signaling.	[383,384,385,386,387,388,389]
	SWAP70	miR-145	tumor suppressor	Decreased growth, migration and invasion.	[390]
	TIAM1	miR-10a/ miR-10b/ miR-22/ miR-29b/ miR-29c/ miR-31/ miR-141/ miR-182/ miR-183/ miR-329/ miR-377	tumor suppressor	Inhibited growth, migration, invasion, EMT, tumor formation and metastasis; Induced apoptosis; Blocked Akt and ERK activation.	[391,392,393,394,395,396,397,398,399,400,401,402,403]
	TIAM1	miR-21/ miR-31	oncogene	Drove migration, invasion, and EMT; Increased B-catenin, vimentin and MMP2 expression.	[404,405]
	VAV2	miR-148a/ miR-149/ miR-195/ miR-331	tumor suppressor	Inhibited spreading, growth, adhesion, migration, invasion, EMT, tumor formation, angiogenesis, and metastasis.	[406,407,408,409]
	VAV3	miR-489/ miR-499	tumor suppressor	Blocked growth, migration and invasion; Induced apoptosis; Promoted sensitivity to chemotherapeutics.	[410,411]
	DOCK1	miR-31/ miR-486	tumor suppressor	Blunted migration, invasion, and metastasis; Suppressed N-cadherin protein expression.	[412,413]
	DOCK3	let-7i/ miR-512	tumor suppressor	Inhibited migration, invasion, adhesion, colonization and metastasis.	[414,415]
	DOCK4	miR-205	tumor suppressor	Reduced growth, migration and invasion; Decreased N-cadherin, vimentin, and ZEB1 protein expression.	[416]
	DOCK6	miR-148b	tumor suppressor	Impaired migration, invasion and metastasis.	[417]
Rho GDIs	ARHGDIA	miR-1/ miR-133a	tumor suppressor	Reversed EMT; Suppressed growth, migration, invasion and metastasis; Inhibited ERK and Akt phosphorylation.	[418,419]
	ARHGDIA	miR-16/ miR-25/ miR-151/ miR-346/ miR-361	oncogene	Promoted growth, migration, invasion, EMT and metastasis; Reduced apoptosis; Activated Akt signaling; Coordinated migration and invasion with FAK.	[288,420,421,422,423,424]
	ARHGDIB	miR-34a	tumor suppressor	Inhibited growth; Induced apoptosis; Enhanced sensitivity to radiation-induced apoptosis.	[420,425]

α-SMA: Alpha smooth muscle actin, AP-1: Activator protein 1, CDKN2BAS: Cyclin dependent kinase inhibitor 2B antisense RNA, ceRNA: Competing endogenous RNA, CSC: Cancer stem cell, CTGF: Connective tissue growth factor, EGFR: Epidermal growth factor receptor, EMT: Epithelial-mesenchymal transition, ERK: Extracellular-signal-regulated kinase, FAK: Focal adhesion kinase, IGF2: Insulin like growth factor 2, LPS: Lipopolysaccharides, LTα: Lymphotoxin-alpha, MAPK: Mitogen-activated protein kinase, MET: Mesenchymal-epithelial transition factor, MMP2: Matrix metallopeptidase 2, MMP9: Matrix metallopeptidase 9, mTOR: Mammalian target of rapamycin, NF-ĸB: Nuclear factor kappa-light-chain-enhancer of activated B cells, PDGFRB: Platelet derived growth factor receptor beta, PEPCK: Phosphoenolpyruvate carboxykinase, PI3K: Phosphoinositide 3-kinase, PTEN: phosphatase and tensin homolog, Rb: Retinoblastoma protein, ROCK1: Rho associated coiled-coil containing protein kinase 1, TGF-β: Transforming growth factor beta, TNFa: Tumor necrosis factor alpha, VEGF: Vascular endothelial growth factor, ZEB1: Zinc finger E-box binding homeobox 1. n/a = not available.

## Abbreviations

3′ untranslated region	3′UTR
cancer stem cell	CSC
diffuse B-cell lymphoma	Dbl
Dbl homology	DH
DOCK-homology region-1	DHR1
DOCK-homology region-2	DHR2
dedicator of cytokinesis	DOCK
extracellular matrix	ECM
epithelial-to-mesenchymal transition	EMT
GTPase-activating protein	GAP
guanine nucleotide exchange factor	GEF
guanine nucleotide exchange modulator	GEM
GDP dissociation inhibitor	GDI
guanosine diphosphate	GDP
guanosine triphosphate	GTP
locked nucleic acid	LNA
messenger RNA	mRNA
microRNA	miRNA/miR
non-coding RNA	ncRNA
pleckstrin homology	PH
phosphate binding loop	P-loop
RNA-induced silencing complex	RISC
small molecule inhibitor of miRNA	SMIR
